# MiR393 and miR390 synergistically regulate lateral root growth in rice under different conditions

**DOI:** 10.1186/s12870-018-1488-x

**Published:** 2018-10-29

**Authors:** Yuzhu Lu, Zhen Feng, Xuanyu Liu, Liying Bian, Hong Xie, Changlun Zhang, Kirankumar S. Mysore, Jiansheng Liang

**Affiliations:** 1 Jiangsu Key Laboratory of Crop Genetics and Physiology/ Key Laboratory of Plant Functional Genomics of the Ministry of Education, Yangzhou, 225009 China; 2 College of Bioscience and Biotechnology, Yangzhou University, Yangzhou, 225009 China; 3 Plant Biology Division, Samuel Roberts Noble Foundation, Ardmore, OK 73401 USA; 4 Department of Biology, Southern University of Science and Technology, Shenzhen, 518055 China

**Keywords:** Auxin, miR390, miR393, *OsTIR1*, *OsTAS3a*, Lateral root growth

## Abstract

**Background:**

Plants have evolved excellent ability of flexibly regulating the growth of organs to adapt to changing environment, for example, the modulation of lateral root development in response to environmental stresses. Despite of fundamental discovery that some microRNAs are involved in this process, the molecular mechanisms of how these microRNAs work together are still largely unknown.

**Results:**

Here we show that miR390 induced by auxin promotes lateral root growth in rice. However, this promotion can be suppressed by miR393, which is induced by various stresses and ABA (Abscisic Acid). Results that miR393 responded to ABA stronger and earlier than other stresses implied that ABA likely is authentic factor for inducing miR393. The transgenic lines respectively over-expressing miR393 and *OsTAS3a* (*Oryza sativa Trans-Acting Short RNA precursor 3a*) displayed opposite phenotypes in lateral root growth. MiR390 was found to be dominantly expressed at lateral root primordia and roots tips while miR393 mainly expressed in the base part of roots at very low level. When miR393 was up-regulated by various stresses, miR390 expression level fell down. However, the risen expression level of miR390 induced by auxin didn’t affect the expression of miR393 and its target *OsTIR1* (*Transport Inhibitor Response 1*). Together with analysis of the two transgenic lines, we provide a model of how the growth of lateral roots in rice is regulated distinctively by the 2 microRNAs.

**Conclusion:**

We propose that miR390 induced by auxin triggers the lateral root growth under normal growth conditions, meanwhile miR393 just lurks as a potentially regulative role; Once plants suffer from stresses, miR393 will be induced to negatively regulate miR390-mediated growth of lateral roots in rice.

## Background

Plant hormones have long been implicated in the coordination of development [1], and no hormone is more ubiquitous as a developmental regulator than auxin [2]. The response of genes to phytohormone is a basic capability for plants to regulate self-growth in order to adapt to environment. Many genes that respond to phytohormones are regulated by microRNA in plants [3–5]. Combined with computational and experimental means, miR393 and miR390 were identified in *Arabidopsis* and rice [6–8] and both are deeply conserved in land plants [9–11], indicating that their biologic roles probably are involved in significant aspects of plants growth and development.

MiR393 molecules were believed to regulate the expression of the auxin receptors of TAAR clade, including *TIR1* (*Transport Inhibitor Response 1*) and its three functional paralogs, AFB1/AFB2/AFB3 (Auxin signaling F-Box proteins 1, 2 and 3) in *Arabidopsis* [12, 13]. Some works in *Arabidopsis* showed that miR393 functions in leaf and root development [13–15], response to stresses [7, 12, 15–17], embryogenic transition [18], and auxin signaling outputs [19]. In rice, over expression of miR393 led to changes in flag leaf angle, plant height, primary root length, seed development and sensitivity to auxin [20], and reduced tolerance to salt and drought [21]. Just recently, OsmiR393 was reported to involve in the process of seed germination and seedling establishment too [22].

MiR390 targets non-coding *TAS3* precursor RNA for the production of a class of another small RNA, ta-siRNA (trans-acting short-interfering RNAs), which cleaves the transcripts of auxin response factors 3/4(ARF3/4) [9, 23–28]. *TAS3* is also highly conserved in various plants [10, 23, 29, 30]. MiR390 and TAS3-ta-siRNAs define a pathway that regulates leaf patterning and developmental timing by repressing the ARF family members ARF2, ARF3, and ARF4 in *Arabidopsis* [26, 27, 31, 32]. MiR390 family in *Arabidopsis* comprises two members, while in rice it contains just one number [8, 33, 34]. MiR390 was also reported to work with miR156 and their targets to control developmental transition in *P. patens* [35].

As described above, the targets’ roles of both microRNAs are related with auxin. Auxin plays central roles in plant growth and development including the regulation of cell division, cell elongation, the establishment of embryo polarity, vascular differentiation, apical dominance and tropic responses to light and gravity [36]. Auxin responses of these diverse developmental events can be regulated at three major steps: auxin metabolism, directional auxin transport and signal transduction [37–41]. Besides the functions mentioned above, it is well known that auxin also plays key roles in the lateral root development [2]. Interestingly, at least part roles of both miR393 and miR390 were regarded as being involved in the development of roots in *Arabidopsis* [14–16, 42, 43], indicating a possibility of crosstalk between them is existed. However, the precise correlation between them is unclear.

Here we elaborately investigated the relationship between miR393 and miR390 in regulating lateral root growth and development in rice. We found that miR393 down-regulates miR390 under ABA (Abscisic Acid) and various stresses, including drought, bacterial, heavy metal, salt, etc. Lateral root growth was triggered by miR390 whose expression was induced by auxin. Together with analysis of transgenic plants, we provided a model of how the 2 microRNAs work together to regulate lateral root growth under both normal and stressed environmental conditions.

## Methods

### Plant materials and growth conditions

Rice cultivar (*Oryza sativa* L. Nipponbare) was used as a control (wide-type) and is the genetic background for all transgenic lines. Surface-sterilized rice seeds were soaked into water for 24 h at room temperature and were germinating under moist conditions (seeds were covered with two layers of moistened cheesecloth) at 37 °C for another 30 h. The germinated seeds were either grown in the petri dishes (diameter: 90 mm) containing N_6_ liquid medium under a 16/8 h photoperiod (approximately 200 μmol m^− 2^ s^− 1^) at 28 °C or grown in the paddy field under natural long-day conditions.

### Stress and hormone treatments

For heavy metal or salt stress, 2-week-old rice seedlings growing in N_6_ liquid medium were transferred into another N_6_ solution containing 200 μM Cu^2+^ (CuSO_4_) or 200 mM NaCl, respectively; For UV light stress, 2-week-old rice seedlings growing in N_6_ liquid medium were shifted with dishes together to incubator illuminating with 100 μmol m^− 2^ s^− 1^ ultraviolet light; For pathogen stress, *Magnaporthe grisea* was incubated into PDA (Potato Dextrose Agar) medium growing to the concentration of 3 × 10^5^ spore ml^− 1^ and then was sprayed onto rice seedlings. The sprayed rice seedlings were kept under moist and dark condition at 26 °C; For drought stress, PEG (polyethylene glycol) infused agar flasks were prepared by using 25% PEG (molecular weight 8000, Sigma, St. Louis) as described previously [44]. Then the 2-week-old seedlings were transferred into flask for further analyzing. The total RNA was extracted from roots of the seedlings exposed to the above stresses after 12 h for RNA analysis. For ABA treatment, seeds or 2-week-old seedlings were transferred into N_6_ solution with 0.5 or 1.0 μM cis, trans-ABA (Sigma Co.) respectively. For IAA treatment, the rice seedlings or seeds were transferred into N_6_ liquid medium containing 10 μM IAA (auxin/indole-3-acetic acid) for designated length of time.

### Northern-blot analysis

Total RNA was extracted from test-ready seedlings and tissues with TRIzol reagent (Invitrogen). The expressions of miR393 and miR390 were detected according to the procedure described by Sunkar et al. [45]. The probes for miR393, miR390 and U6 were DNA oligonucleodes synthesized by ShengGong Co., Shanghai, China. The DNA oligonucleodes of 5′- AAGGGGTGACCTGAGAACACA-3′ served as probe for miR393, 5’-GGCGCTATCCCTCCTGAGCTT-3′ for miR390, 5’-ATTTCTCGATTTGTGCGTGTC-3′ for U6; The three probes were labeled with γ-^32^P-ATP at 5′ terminal by using phosphatase and T4 Polynucleotide Kinase. Total RNA (about 50 μg) was loaded onto warmed 15% polyacryl-amide TBE-Urea gels and run at 150 V in 0.5 × TBE for 1.5–2 h. Small RNAs were electro-blotted to a Hybond N^+^ membrane (Amersham Pharmacia Biotech, UK) for 30 min at 24 V in 0.5× TBE buffer. Membranes were hybridized overnight at room temperature with 100 ng/ml miRNA probe labeled with γ-^32^P-ATP. Membranes were washed twice for 5 min each with 2 × SSC, 0.1% SDS at room temperature and twice for 15 min each with 2× SSC, 0.1% SDS at 50 °C and then were autoradiographed.

### Construction of expression vector and generation of transgenic rice lines

To over express miR393 in rice, the expressional vector pCAMBIA1301 that has a constitutive promoter *Ubi1*, which is very effective for monocotyledons, was available. A DNA fragment of miR393a’s precursor with a length of 154 bp was cloned by PCR with primers (Forward: 5′- AAGGATCCAGCAGCAATGTCTTGGGGAA-3′; Reverse: 5′- GCGAGCTCTTTAATGGCTAGAGGAAGCC-3′). To over express *OsTAS3a* (Gene ID: EU293144), we amplified the full gene’s sequence with the indicated primers (Forward: 5′-AAGGATCCACCCCCCTTTTCTTCTTCTT-3′; Reverse: 5′- GCGAGCTCTTAGGATCAATAAAACAAGG-3′). To clone the promoters of OsmiR393a and OsmiR390, the primers for the promoter of miR393a (Forward: 5’GGAAGCTTGCATCTGGTGATCACACACA-3′; Reverse: 5’-AACCATGGCACCGGCTGGCCCTTCTCTC-3′) and for that of miR390 (Forward: 5’-GGAAGCTTAGCCACACAACAAGCTACTC-3′; Reverse: 5′- AACCATGGTCTCTCCCTTTGAACGCCTA-3′) were used. All cloned PCR products were reclaimed and cloned into pUC18 and verified by sequencing. The DNA fragments of miR393a and *OsTAS3a* driven by *Ubi1* promoter were constructed into pCAMBIA1301. To construct reporter gene fusions, the original 35S promoter of GUS in pCAMBIA1301 was replaced by promoters of miR393 and miR390, respectively. All the constructed expression vectors were introduced into rice calli through *Agrobacteriu* (EHA105) mediated methods [46].

### GUS activity and staining assays

For histochemical analysis, different tissues were stained with X-Gluc (5-brom-4-chloro-3-indolyl glucuronide) as described previously [47]. GUS activity was analyzed in total proteins by a fluorescence using 4-methylumbelliferyl glucuronide as described by Jefferson [48]. The fluorescent product 4-methylumbelliferone (MU) was quantified using a fluorometer. Standard solutions of MU in 0.2 M Na_2_CO_3_ were used for calibration. Total proteins were extracted with buffer (50 mM sodium phosphate, pH 7.0, 1 mM EDTA, 0.1% [*v*/v] Triton X-100, and 10 mM 2-mercaptoethanol). The fluorogenic reaction was performed in 1 mL volume with 1 mM 4-methylumbelliferyl-b-D-glucuronide (Invitrogen) in the extraction buffer supplemented with a 0.1 mL aliquot of protein extract supernatants. GUS activity was calculated as picomoles MU per minute per milligram of protein.

### Quantitative RT-PCR

For quantitative RT-PCR analysis of *OsTIR1* (Gene ID: Os05g05800) and *OsARF3* (Gene ID: Os05g48870), 2 μg of total RNA was reversely transcribed in a total volume of 20 μL with 0.5 mg oligo (dT)15, 0.75 mM dNTPs, 10 mM DTT, and 100 U SuperScript II RNase H2 reverse transcriptase (Invitrogen). For quantitative RT-PCR of OsmiR390 (Accession number: MI0001690), OsmiR393a (Accession number: MI0001026) and OsmiR393b (Accession number: MI0001148), 3′ reverse primers of the three precursors were firstly used for reversely transcribing in 10 μg of total RNA. PCR was performed in a total volume of 20 μL with 1 μL of the RT reactions including a CFX real-time PCR instrument (Bio-Rad) and SYBR Green mixture (Roche), 0.2 mM gene-specific primers, and 1 U Taq Polymerase (TaKaRa). The primers for quantitative RT-PCR are listed as the following: OsmiR390 RTF: 5′-GGAGAGATGTTTTGAGGAAGGG-3′, OsmiR390 RTR: 5′-ATTTAATTGGTCGTGTGGTAAG-3′; OsmiR393a RTF: 5′-AGCAGCAATGTCTTGGGGAA-3′, OsmiR393a RTR: 5′-TTTAATGGCTAGAGGAAGCC-3′; OsmiR393b RTF: 5′-TCGGCCTGAGGAAACTAGTGG-3′, OsmiR393b RTR: 5′-GAAGATGAGGAGGCGGAAGCA-3′; *OsTIR1* RTF: 5′-CATGCTGATCGTCTTGAGATGC-3′, *OsTIR1* RTR: 5′-GTCTCCAGCTTTGCTGCGTTC-3′; *OsARF3* RTF: 5′-CCACTCCAGCCTTATCCTAC-3′, *OsARF3* RTR: 5′-GCTGGAACCTTCTCAGTCAAAG-3′. The lengths of all fragments amplified by PCR are in a range of 127 to 164 bp. A total of 28 to 30 cycles was performed. The expression levels of the samples were normalized by *OsUbiquitin* gene (Forward: 5’-AACCAGCTGAGGCCCAAGA-3′, Reverse: 5’-AACCAGTCCATGAACCCGG-3′). Experiments were performed with three biological replicates, of which each was performed in three technical replicates.

## Results

### Expression patterns of miR390 and miR393 in rice

**Fig. 1:**
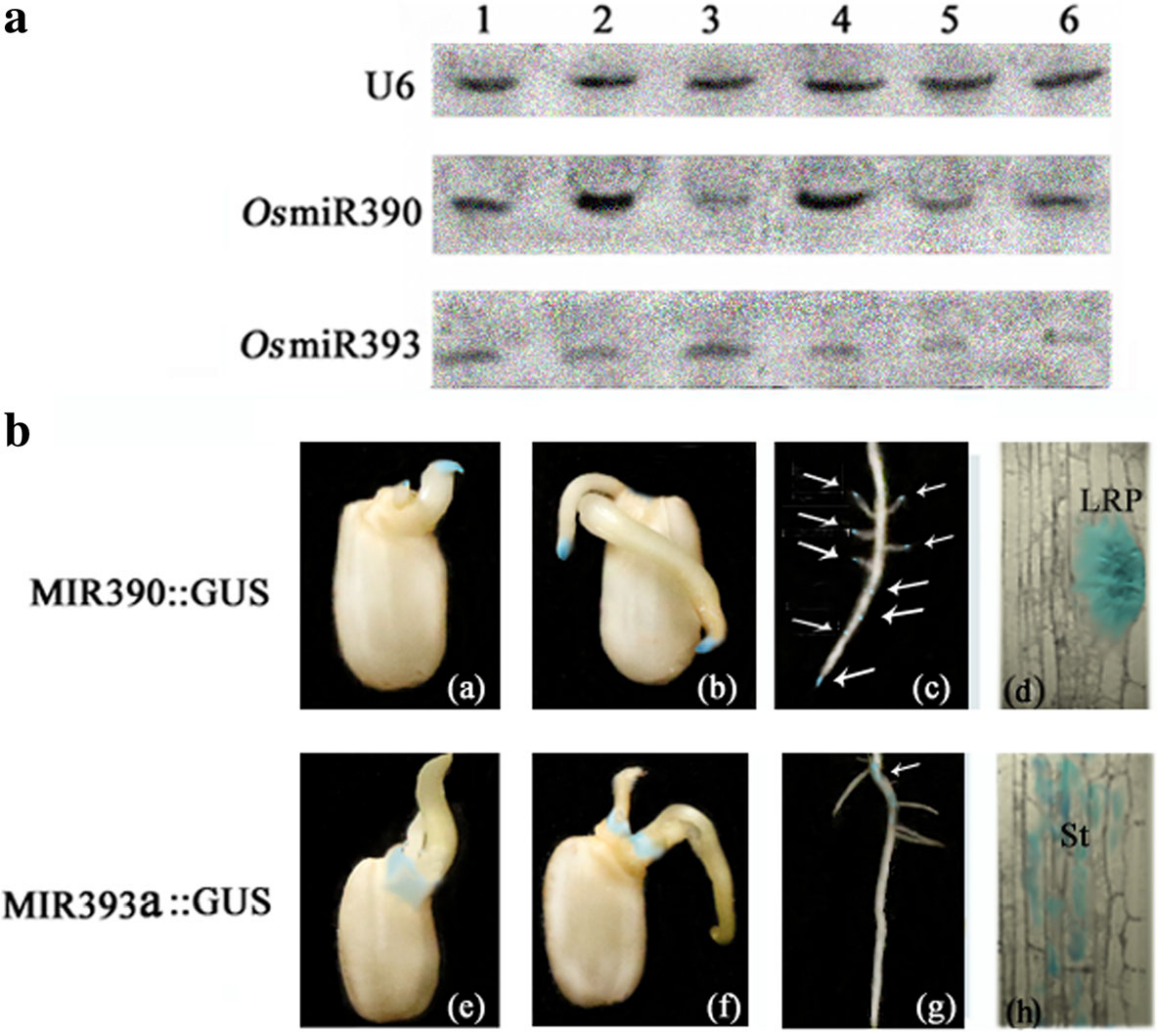
The expression patterns of miR393 and miR390 in rice. (**a**) Tissue expression pattern. For small RNA gel blots, 50 μg of total RNA was loaded and the blots were probed for miR390, miR393 and U6 RNA (as a loading control). The lanes represent the tested tissues of 2-week-old seedlings (lane1), lateral root primordia (lane 2), the base part of root (lane 3), 5-mm-long roots tips of 4-week-old-seedlings (lane4), the base part of shoot in 4-week-old-seedlings (lane 5), and flowers (lane 6), respectively. (**b**) GUS staining pattern of the transgenic rice MIR390::GUS and MIR393a::GUS. GUS is visualized in blue. The staining is observed in the 3-d-germinated seeds (a and e), 5-d-germinated seedlings (b and f), primary root of 3-week-old seedlings (c and g). semi-thin slices of 2-week-old primary root (d and h). LRP: lateral root primordia; St: stele, Bars = 2 cm [(a), (b), (c), (e), (f), (g)]; Bars = 50 μm [(d) and (h)].

Considering some clues that revealed part roles of miR393 and miR390 are involved in the development of roots in *Arabidopsis* [14–16, 42, 43] and the facts that the targets of the 2 microRNAs are associated with Auxin [6–8], we speculated there might have some crossed correlation between the 2 microRNAs in regulating root growth and development. To validate this hypothesis, we investigated the expressional pattern of the two microRNAs in rice because understanding the spatial and temporal dynamics of a miRNA is a key to understand miRNA’s function. By northern blots, we detected the expression of both miR390 and miR393 in different tissues, including 2-week-old seedlings, lateral root primordia, root tips, old roots, old shoots and flowers. We found that miR390 was highly expressed in lateral root primordia and root tips while moderately expressed in 2-week-old seedlings and lowly expressed in other tissues (Fig. 1a). However, the RNA abundance of miR393 was much lower in most tested tissues except for the base part of roots with slight stronger intensity (Fig. 1a). To further analyze the spatial and temporal expression pattern of the two microRNAs, an ~ 2-kb putative promoter sequences upstream of the predicted fold-back structure of both miR390 and miR393a were isolated and fused to the coding region of β-glucuronidase (GUS) to generate promoter-GUS transgenic plants. Then the expression constructs were introduced into rice calli by *Agrobacterium* (EHA105) transformation. Usually during the process of rice germination, the primary root firstly emerges after about 3 days of incubation and then the shoot tip appears 2 days later. Staining assay of the transgenic plants showed that a striking GUS activity of miR390 in the primary root tips was firstly observed after 3 days of incubation, while a weaker GUS activity of miR393 just emerged at the base of the primary root [Fig. 1b. (a) and (e)]. After 7 days of germination, other than appearing in the primary root tip and shoot apical meristem, the GUS staining of miR390 also started to appear in the first lateral root tip while the staining of miR393 was only slightly emerging around region of the scutellar epithelia [Fig. 1b. (b) and (f)]. GUS assay of the 3-week-old primary root showed that the staining of miR390 was mainly emerging in the tips of the both primary root and lateral roots as well as the places where the lateral roots initiate from [Fig. 1b. (c)]. However, GUS staining of miR393 was merely observed in the base part of the primary root where the lateral roots had well developed [Fig. 1b. (g)]. To observe how the two genes express in the microstructure of root, 2-week-old primary root was sliced and GUS staining assay was performed. A strong GUS staining of miR390 was clearly observed in lateral root primordia, while only some scattered GUS spots of miR393 emerged in the base part of the primary root [Fig. 1b. (d) and (h)]. Overall, no overlap of GUS staining between miR390 and miR393 was observed in the detected materials, hinting a likely antagonistic relationship of the two microRNAs probably was existed.

### MiR390, not miR393, responds to auxin in rice

**Fig. 2:**
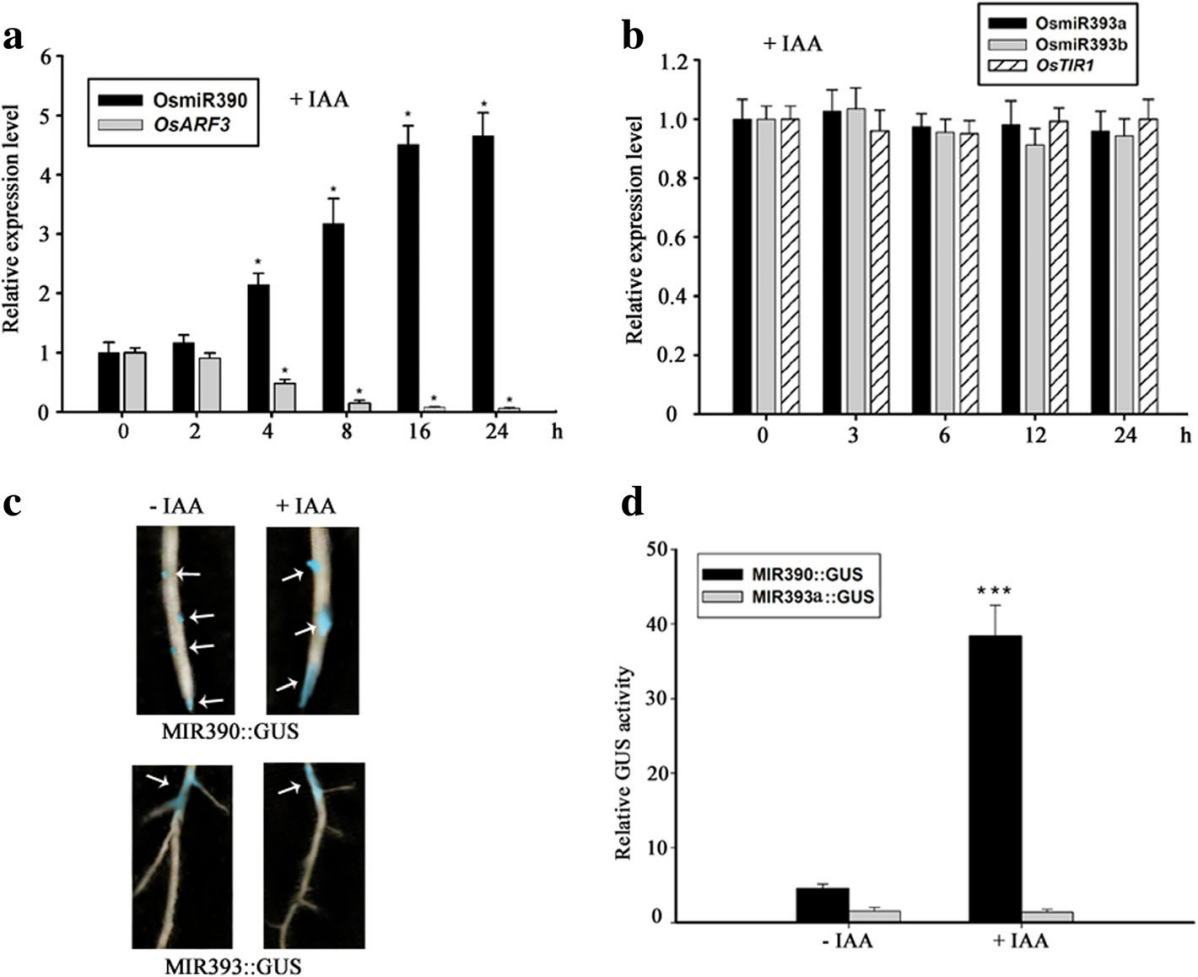
Response of miR390/*OsARF3* and miR393/*OsTIR1* to auxin in rice. **a** Time course analysis of expression of OsmiR390 and *OsARF3* in response to AUX/IAA (auxin/indole-3-acetic acid). 2-week-old seedlings were exposed to 10 μM IAA for designed time. Expression was analyzed by qRT-PCR. *, Significant difference at *P* < 0.05 compared with No treatment by Student’s t-test (*n* = 3; means ± SDs). **b** Time course analysis of expression of OsmiR393a, OsmiR393b and *OsTIR1* in response to AUX/IAA (auxin/indole-3-acetic acid). 2-week-old seedlings were exposed to 10 μM IAA for designed time. Expression was analyzed by qRT-PCR. (n = 3; means ± SDs). **c** GUS staining assay of response of OsmiR390 and OsmiR393 to IAA. GUS staining assay of primary root in MIR390::GUS and MIR393a::GUS was performed after being exposed to 10 μM IAA for 12 h. GUS staining is pointed by white arrows. **d** Relative GUS activity of MIR390::GUS and MIR393a::GUS in response to IAA. ***, *P* < 0.001; Student’s t-test

Even though the targets of both miR390 and miR393 are believed to be involved in auxin signaling cascade in *Arabidopsis* [6, 7, 12, 13, 42, 43], it yet remained unclear what the relationship between the two microRNAs was. To test whether the two microRNAs and their targets respond to the auxin in rice, the roots of 2-week-old seedlings were exposed to 10 μM IAA for time course analysis by qRT-PCR (quantitative Reverse-Transcription PCR). Results showed that the expressional level of OsmiR390 started to rise up after 2 h exposure and reached to peak after 16 h of treatment (Fig. 2a). Meanwhile, an opposite expression pattern of miR390’s target *OsARF3* was observed during the process (Fig. 2a). However, the expression level of OsmiR393a, OsmiR393b (rice have two miR393 members) and its target *OsTIR1* always kept constant under the treatment (Fig. 2b).

To further investigate the response of the two microRNAs to auxin, GUS staining assay of both MIR390::GUS and MIR393a::GUS was performed in the roots with/without IAA treatment. An apparent increment of GUS staining in the region of lateral root primordia was observed in MIR390::GUS after being exposed to IAA for 12 h (Fig. 2c). While the GUS staining of miR393 which mainly appeared at the older parts of roots didn’t change under IAA treatment (Fig. 2c). We further tested GUS activity from the whole roots of 2-week-old seedlings of the two types of transgenic lines. Results showed that the GUS activity dramatically increased in MIR390::GUS after 12 h treatment, while the activity of MIR393a::GUS had no apparent change (Fig. 2d).

### MiR393 up-regulated and miR390 down-regulated by various stresses

**Fig. 3:**
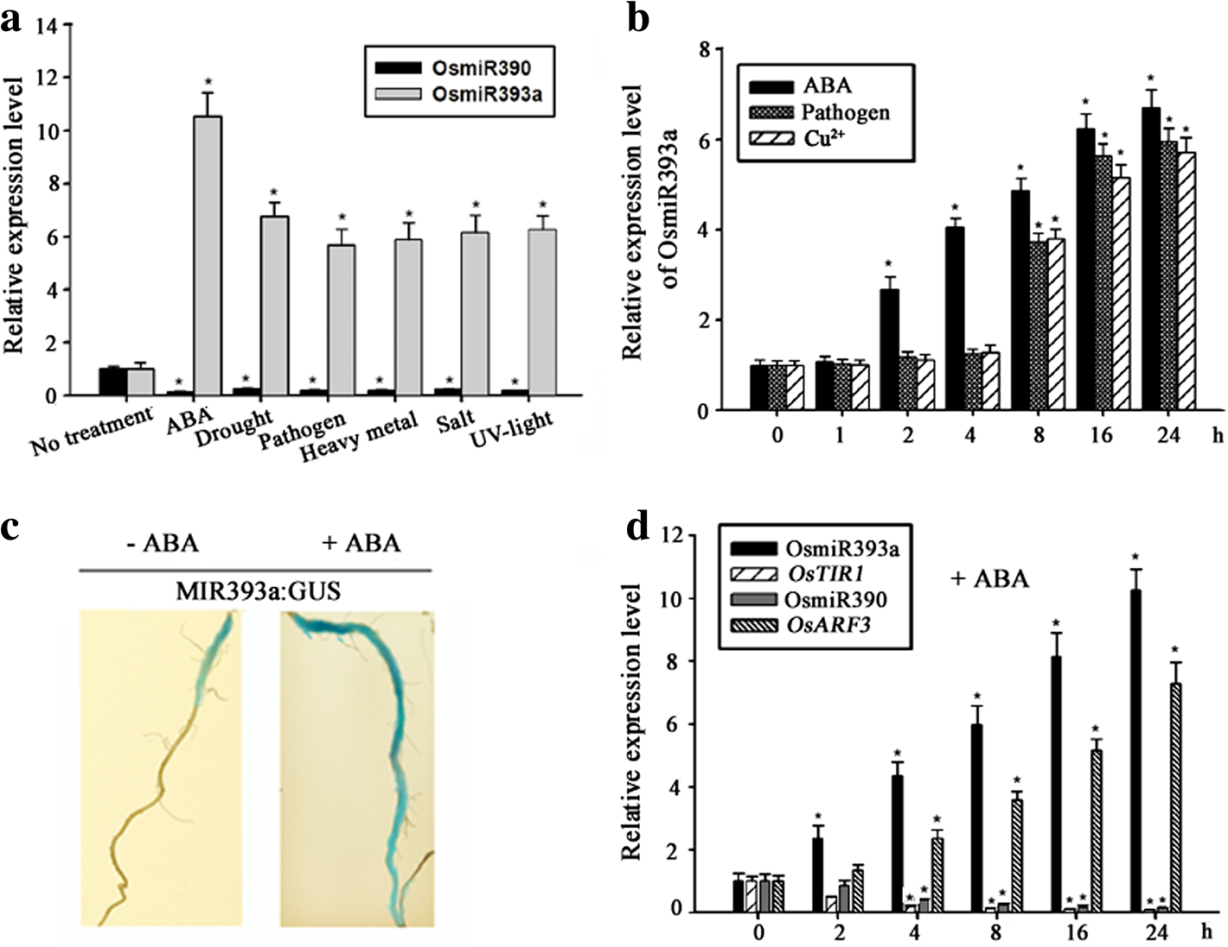
The expression of miR393 is induced by diverse stresses in rice. **a** The expression of miR393a and miR390 in response to ABA and stresses. The RNA levels were detected by qRT-PCR in 2-week-old seedlings exposed to ABA (1.0 μM) and different stresses for 12 h, including drought (25% PEG), pathogen (*Magnaporthegrisea*), Cu^2+^ (200 μM), UV (100 μmol m^− 2^ s^− 1^ ultraviolet), NaCl (200 mM). 2-week-old seedlings without stress treatment served as control. *, Significant difference at P < 0.05 compared with No treatment by Student’s t-test (n = 3; means ± SDs). **b** Time course analysis of miR393a levels in response to ABA (1.0 μM), pathogen (*Magnaporthegrisea*), and Cu^2+^ (200 μM) by qRT-PCR. *, Significant difference at P < 0.05 compared with No treatment by Student’s t-test (n = 3; means ± SDs). **c** GUS staining analysis of spatial expression pattern of miR393a under ABA treatment for 12 h in the transgenic plant MIR393a::GUS. The 2-week-old primary roots were stained before and after treatment respectively. Bar = 2 cm. **d** Time course analysis of miR393a, *OsTIR*, miR390, and *OsARF3* levels in response to ABA. 2-week-old seedlings were incubated into N_6_ liquid medium containing 1 μM ABA. Expression was analyzed by qRT-PCR. *, Significant difference at P < 0.05 compared with No treatment by Student’s t-test (n = 3; means ± SDs)

In *Arabidopsis*, some clues from several reports indicated that, at least partial, the roles of miR393 are tied with the environmental conditions [7, 12, 15–17]. But whether the change of miR393 affects the expression of miR390 yet remained unknown. To investigate whether and how miR393 and miR390 respond to both biotic and abiotic stresses in rice, the expression of the 2 microRNAs was quantified under different stresses by qRT-PCR. When the 2-week-old seedlings were exposed to ABA and various stresses for 12 h, including bacterial, drought, Cu^2+^, UV, and NaCl, the expression of OsmiR393a apparently increased, especially under ABA treatment (Fig. 3a). However, the expression of OsmiR390 was down-regulated by all these treatments and ABA (Fig. 3a). Time course analysis showed that miR393 started to respond to ABA at point of 2-h-treatment while it started to responded to pathogen (*Magnaporthegrisea*) and high concentration of Cu^2+^ at point of 8-h-treatment (Fig. 3b). The long span between the response to ABA and other stresses implied that ABA probably was the direct factor that caused the rise of miR393’s expression. It is well known that various stresses can cause rise of ABA in plants and that ABA, as one kind basic phytohomone, impacts on many genes’ expression [49].

GUS staining assay in MIR393a::GUS showed that area of GUS staining expanded from base part to almost whole root and that the intensity was also strikingly strengthened after roots were exposed to ABA for 12 h (Fig. 3c). It is well known that microRNA negatively regulated its targets. Time course analysis of the 2 microRNAs and their targets by qRT-PCR under ABA treatment showed that OsmiR393a gradually rose up and its target OsTIR1 gradually declined down, meantime, OsmiR390 was down-regulated while *OsARF3* was up-regulated gradually (Fig. 3d).

### Overexpressing miR393 displayed a variety of growth defects including a reduced number of lateral roots

**Fig. 4:**
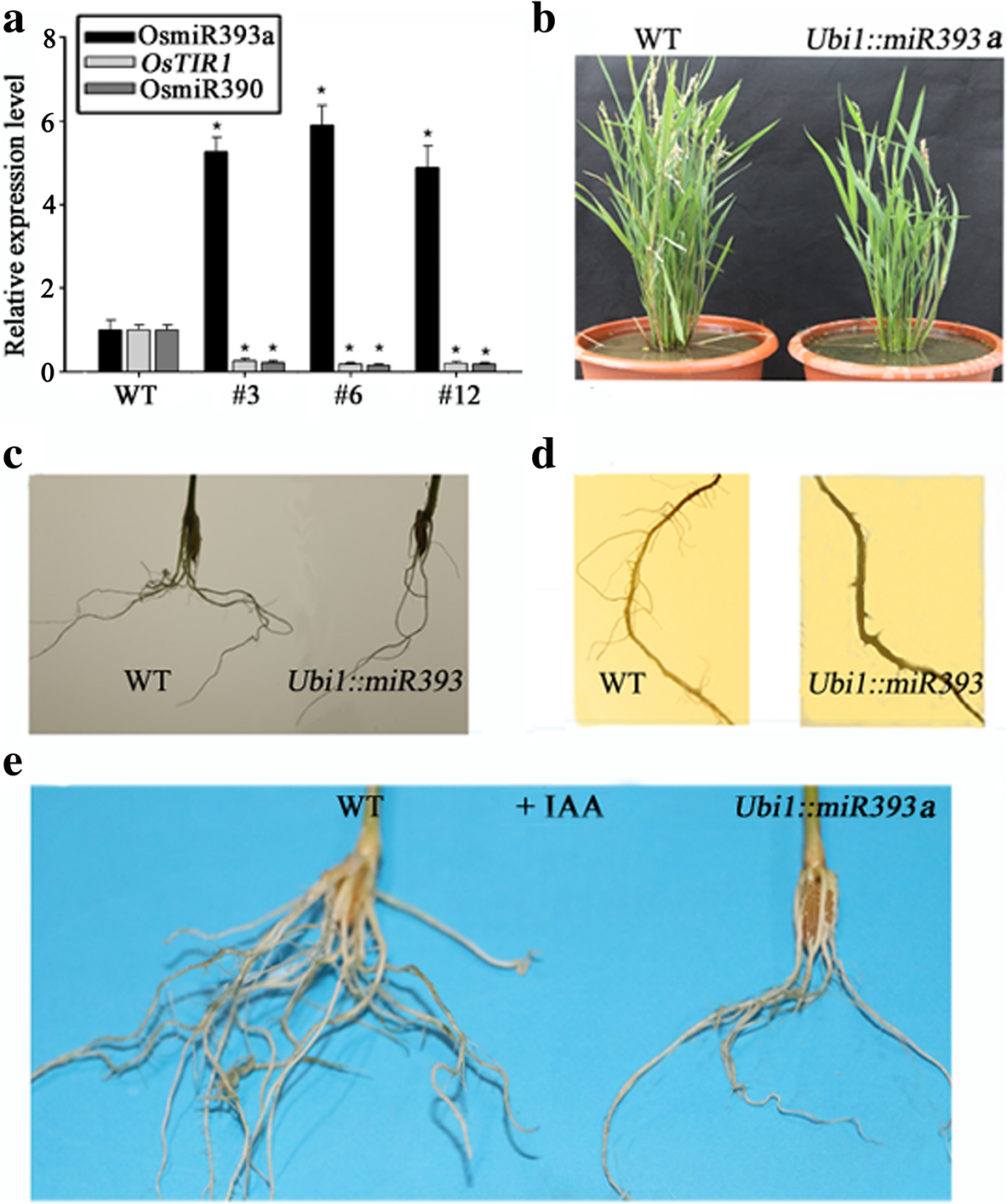
The phenotype of *Ubi1::miR393*. **a** Identifying the transgenic plants. The expression of OsmiR393a, *OsTIR1* and miR390 was measured by qRT-PCR. “#-number” represents different transgenic lines. *, Significant difference at P < 0.05 compared with the wild type by Student’s t-test (n = 3; means ± SDS). **b** Comparison of adult phenotypes between the wild type and *Ubi1::miR393a*. Bars = 50 cm. **c** Comparison of the root systems of 2-week-old seedlings between the wild type and *Ubi1::miR393a*. Bars = 2 cm. **d** Comparison of the primary roots of 4-week-old seedlings between the wild type and *Ubi1::miR393a*. Bars = 5 mm. **e** Comparison of the root systems between the wild type and *Ubi1::miR393* after their seeds geminating and growing in N_6_ liquid medium with 10 μM IAA for three weeks. Bars = 2 cm

To investigate how miR393 affects the growth development in rice, we generated transgenic rice overexpressing miR393. The precursor of *OsmiR393a* comprised a length of ~ 150 bp was cloned and constructed into expression vector driven by *Ubiquitin1* (*Ubi1*)promoter. Then this constructed vector was brought into rice genome by *Agrobacterium*-mediated transformation. After harvesting the seeds of positive T_0_ plants, we selected transgenic seeds by *hygromycin-*resistant segregation and surveyed their growth traits in T_1_ generation. The expression of miR393/*OsTIR1* in T_1_ transgenic plants was firstly quantified by qRT-PCR. MiR393a’s expression was found to be notably increased while *OsTIR1*’s expression was decreased accordingly in several transgenic lines (serial number as #3, #6, #12) (Fig. 4a). Overall, the phenotype of *Ubi1::miR393a* presented as multiple defects, such as a tardy growth, slightly shorter height, lower yields, and less tillers (Fig. 4b), etc. The final height of *Ubi1::miR393a* was about 80% of the wild-type. When the ear of wild-type rice became matured, the ear of *Ubi1::miR393a* was still green (Fig. 4b). The lifetime of *Ubi1::miR393a* was about 15 days longer than the wild type. Compared with wild-type plants, *Ubi1::miR393a* had much fewer lateral roots (Fig. 4c and d). The number of lateral roots of *Ubi1::miR393a* were just about half of the wild type. However, the length of primary roots between the two phenotypes had no significant difference (Fig. 4c). Furthermore, the primary root of the wild type had more root hairs than *Ubi1::miR393a* (Fig. 4d). It is well known that auxin can stimulate lateral root growth, but the growth of lateral root in *Ubi1::miR393a* was insensitive to IAA. When the seeds of the two phenotypes exposed to IAA for 3 weeks, the wild type produced more lateral roots while *Ubi1::miR393a* had no apparent variety of number of lateral roots (Fig. 4e), suggesting the possibility that lateral root growth mediated by auxin probably is through pathway of miR393’s targets.

### MiR390 functions downstream of miR393

**Fig. 5:**
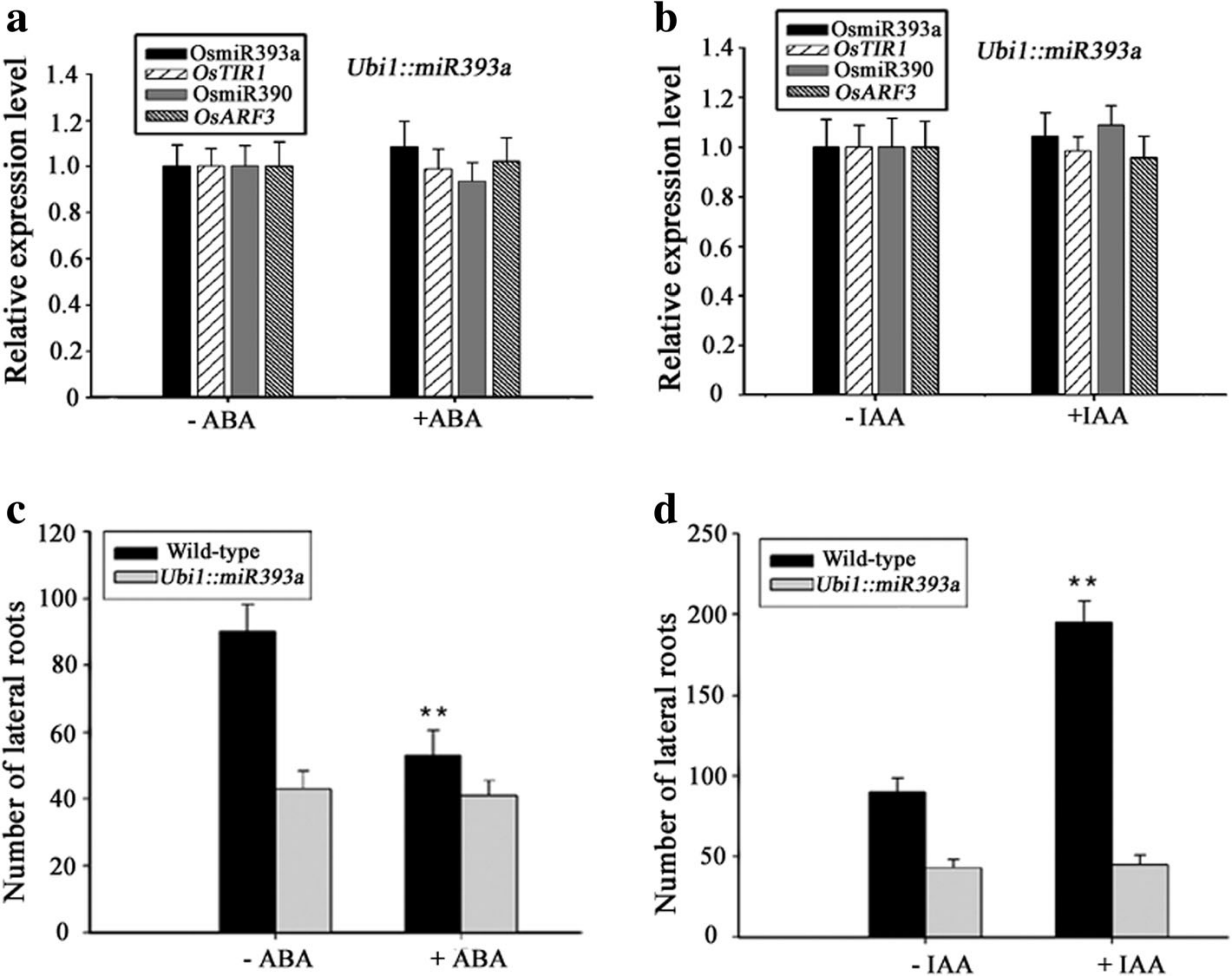
Effects of ABA and IAA on *Ubi1::miR393a* and the wild type. **a** The response of OsmiR393a and OsmiR390 as well as their targets to ABA in *Ubi1::miR393a*. 2-week-old seedlings was exposed to N6 liquid medium with 1 μM ABA for 12 h and expression was analyzed by qRT-PCR (n = 3; means ± SDS). **b** The response of OsmiR393a and OsmiR390 as well as their targets to IAA in *Ubi1::miR393a*. 2-week-old seedlings was exposed to N6 liquid medium with 10 μM IAA for 12 h and expression was analyzed by qRT-PCR (n = 3; means ± SDS). **c** Effects of ABA on lateral root growth of the wild type and *Ubi1::miR393a*. The seeds of the wild type and *Ubi1::miR393a* was incubated into N_6_ liquid medium with 0.5 μM ABA for two weeks. **,P < 0.001; Student’s t-test. **d** Effects of IAA on lateral root growth in the wild type and *Ubi1::miR393*. The seeds of the wild type and *Ubi1::miR393a* was incubated into N6 liquid medium with 10 μM IAA for two weeks. **,P < 0.001; Student’s t-test

Even though Fig. 3a and d showed that miR393 and miR390 have opposite ways in response to ABA and various stresses, but it was still unclear if the 2 types of response are in the same pathway. To address this question, we investigated how miR393, miR390 and their targets responded to ABA and IAA in *Ubi1::miR393a* lines, in which its target *OsTIR1* was inhibited (Fig. 4a). Compared to the wild type, the expression level of miR390 was significantly lower in *Ubil1::miR393a* (Fig. 4a), indicating that over-expression of miR393 can suppress the expression of miR390. Then we exposed both the wild-type and *Ubi1::miR393a* to ABA and IAA for 12 h, respectively. Expression analysis by qRT-PCR showed that miR390 didn’t respond to ABA in *Ubil1::miR393a* (Fig. 5a), suggesting that down-regulated expression of miR390 in the wild type by various stress and ABA was caused by up-regulated expression of miR393 (Fig. 3a and d). Meantime, miR390 didn’t respond to IAA in *Ubi1::miR393a* too (Fig. 5b), suggesting that the response of miR390 to auxin (Fig. 2) probably need the roles of miR393’s targets.

To further investigate the effects of ABA and IAA on lateral root growth in the two genotypes, the seeds of the wild type and *Ubi1::miR393a* were incubated into N6 liquid medium containing 0.5 μM ABA and 10 μM IAA respectively for germinating. The number of lateral roots was markedly decreased by ABA after 2 weeks treatment in the wild type, while the number of lateral roots was not been apparently affected in *Ubi1::miR393a* (Fig. 5c). Meanwhile, the number of lateral roots of the wild type markedly increased by IAA after 2 week treatment, while the number of lateral roots of *Ubi1::miR393a* didn’t changed evidently (Fig. 5d). These results indicated that the effects of ABA and IAA on the lateral root growth probably are through pathway of miR393.

### Over-expressing *OsTAS3a* displayed a phenotype of denser lateral roots in rice

**Fig. 6:**
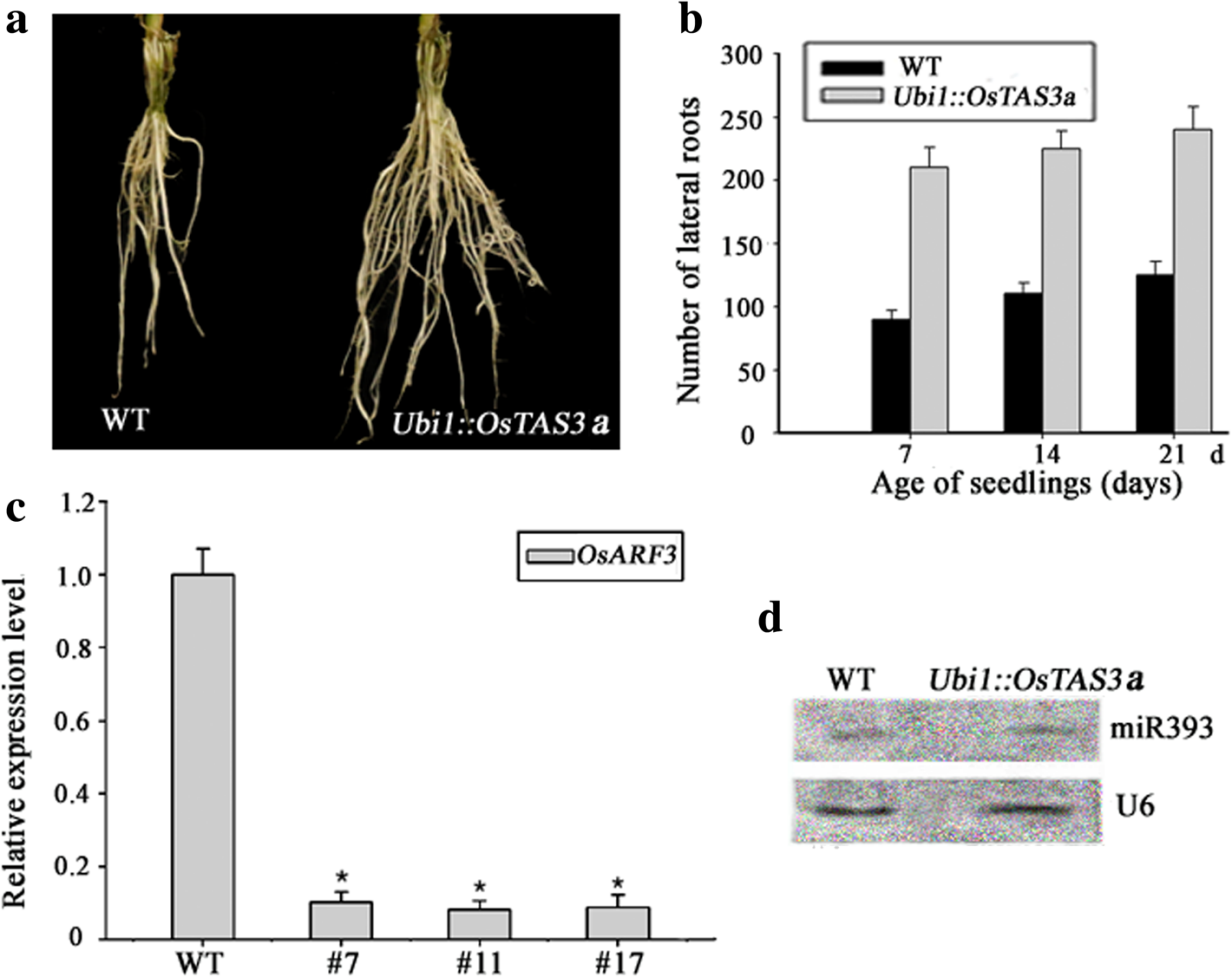
The phenotype of transgenic lines over-expressing *OsTAS3a.* **a** The phenotype of root systems in 2-week-old seedlings of the wild type and *Ubi1::OsTAS3a*. Bar = 2 cm. **b** The number of lateral roots of the wild type and *Ubi1::OsTAS3a.* Statistics were from seedlings at age of 7th, 14th, and 21st day. **c** The expression of *OsARF3* in the wild type and *Ubi1::OsTAS3a*. Expression was analyzed by qRT-PCR. “#-number” represents different transgenic lines. *, Significant difference at P < 0.05 compared with the wild type by Student’s t-test (n = 3; means ± SDS). **d** The expression of miR393 in the wild type and *Ubi1::OsTAS3a*. RNA analysis was performed by northern blot

The mechanism of producing second class of endogenous small RNA (ta-siRNA: trans-acting short-interfering RNAs) by microRNA might be a process of cascade amplification [9, 23, 28]. It is well known that miR390 targets non-coding *TAS3* precursor RNA for the production of ta-siRNA which cleaves the transcripts of auxin response factors (ARF3/4) [9, 23–27]. In rice, there are 3 *OsTAS3* members (*OsTAS3a, OsTAS3b, OsTAS3c*) targeted by miR390 and each kind of TAS3-derived ta-siRNAs have their own target of ARF genes [3]. To avoid lethal outcome of overexpression of miR390, we overexpressed the *OsTAS3a* gene driven by *Ubiquitin1* (*Ubi1*)promoter in rice with anticipation of getting a weak substitution of miR390 overexpression. The transgenic lines overexpressing *OsTAS3a* were generated by *Agrobacterium*-mediated transformation and were identified by qRT-PCR (Fig. 6c). *OsARF3–1*(Os05g48870), targeted by *OsTAS3a-*siRNA [3, 50], was dramatically down-regulated in several T_1_ transformants (serial number as #7, #11, #17) (Fig. 6c). Most positively transgenic lines display a phenotype of denser root system in T_1_ generation (Fig. 6a). The number of lateral roots of *Ubi1::OsTAS3a* was about 2 times of wild-type at different stages (Fig. 6b). To test if overexpression of *OsTAS3a* affected the expression of miR393, the expression levels of miR393 was detected by northern blots. Results showed that the expression of miR393 was not altered between wild-type and *Ubi1::OsTAS3a* (Fig. 6d), further suggesting that miR390/*OsTAS3a*/*OsARF3* work downstream of miR393.

## Discussion

Despite of some studies revealed that miR390 and miR393, at least partial roles, were involved in root growth and development in *Arabidopsis* [14–16, 42, 43], few relevant roles of the 2 microRNAs were reported in rice. In this work, we investigated how the distinctive roles of the two microRNAs are allocated and cooperated in response to environmental conditions to regulate lateral root growth. *Ubi1::miR393a* displayed a reduced number of lateral roots (Fig. 4c), while *Ubi1::OsTAS3a* (logically approximate to overexpression of miR390) exhibited an increased number of lateral roots (Fig. 6a), indicating that the roles of the two microRNAs are opposite to each other in regulating lateral root growth.

**Fig. 7:**
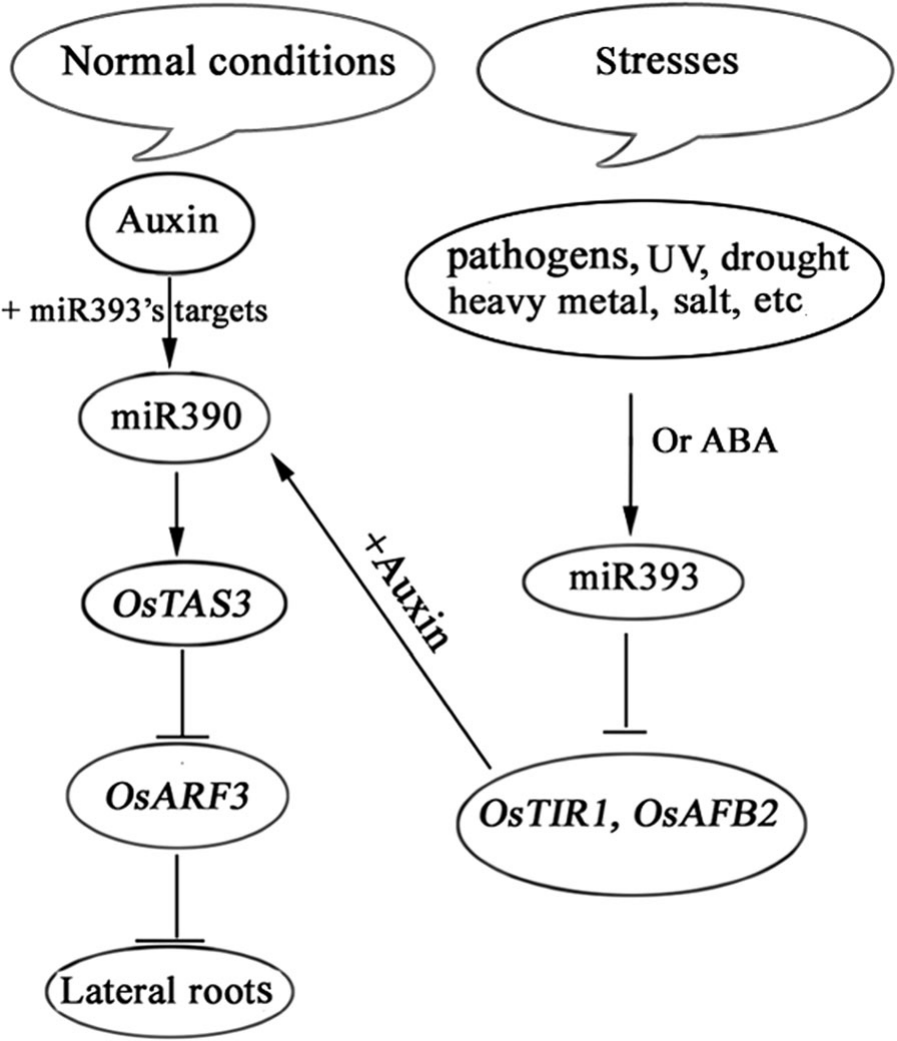
Model for the coordinated roles between miR393 and miR390 in regulating lateral root growth. Lateral root growth is mainly promoted by risen-expression of miR390 controlled by auxin under normal conditions, meanwhile miR393 just lurks as a potentially regulative role; When plants are exposed to ABA and various stresses, miR393 is then induced to suppress lateral root growth by inhibiting the expression of miR390

The expression of OsmiR393 was not changed in the *Ubi1::OsTAS3* (Fig. 6d), while the expression of miR390 was suppressed in *Ubil::miR393a* (Fig. 3a and Fig. 5a). These results combined with the results from Fig. 2 and Fig. 3a and d, further indicated that miR390 functions downstream of miR393. Our results here suggested a model of how the two microRNAs work together to regulate lateral root growth in rice under different environmental conditions (Fig. 7). In this model, we suggest that miR390 promoting lateral root growth under normal environmental conditions is through way of perceiving auxin (Fig. 2). However, the response of miR390 to auxin may need miR393’s targets because miR390 can’t respond to auxin in *Ubi1::miR393a* whose *OsTIR1* was inhibited (Fig. 5b). In addition, the induction of miR390 mediated by IAA didn’t change the expression of miR393/*OsTIR1* (Fig. 2b), implying that miR393’s targets regulating miR390 probably need to combine auxin.

### MiR390 promoting lateral root growth likely is one of branches regulated by miR393/targets

Auxin has versatile roles in regulation of plant growth not only involved in lateral root growth [2, 36]. Regarding that miR393’s targets are receptor of auxin [12, 13], it is reasonable to believe that miR393/targets locating at upstream of auxin cascades might have more roles than just regulating lateral root growth. The phenotype of *Ubi1::miR393a* that displayed multiple-defects including a tardy growth, slightly shorter height, lower yields, and less tillers (Fig. 4b), indicated miR393/targets might have a broad range of roles in regulation of plant growth. This also consisted with the previous reports that miR393 involved in various aspects of plant development [7, 12–16, 18]. By contrast to miR393, the role of miR390 likely is relatively specific in just regulating lateral root growth. The phenotype of *Ubi1::OsTAS3*, in which *OsARF3* was inhibited, mainly displayed an increased number of lateral roots without other apparent difference (Fig. 6a). The specific expression patter of miR390 which mainly focus on the site of lateral root primordia and root tips (Fig. 1b) further indicated the relatively specific role of miR390. So, miR390 likely is just one of branches regulated by miR393/targets.

### The coordinated roles between miR390 and miR393 in regulating lateral root growth likely are very subtle and elaborative for plants to adapt to changing environmental conditions

When plants are subjected to severe stresses, some stress-related genes will rapidly alter their expression for survival [1]. The networks of fluctuation between different genes usually are complicated. Our studies showed that the expression level of miR390 is sensitively regulated by miR393 which can be also adaptively regulated by environmental conditions (Fig. 3a and b). Meanwhile similar concerted expressional fluctuations between their targets were also clearly manifested under ABA treatment (Fig. 3d). Figure 3d clearly showed the tested genes’ expression levels exhibited well synchronous fluctuation with each other, indicating that a subtle and elaborative coordination exists between 2 microRNAs and their targets. Despite that all stresses tested here can induce the expression of miR393, the results that miR393 can be induced stronger and earlier by ABA than other stresses implied that ABA probably is the direct factor for rising expression levels of miR393. An indirect evidence that the expression of miR390 is up-regulated in a rice ABA deficient mutant, *Osaba1* [51], further support our hypothesis. Moreover, highly conserved traits of the 2 microRNAs and their targets in both monocotyledons and dicotyledons suggest that their coordinated roles involved in lateral root growth between them might be conserved in high plants..

## Conclusions

Based on the above results and discussions, we conclude that under normal conditions miR393 likely lurks as a potentially regulatory role due to its very low expression levels in various tissues. Only are plants exposed to stresses, miR393 is then up-regulated to suppress the expression of miR390 and thus resulting in suppression of lateral root growth. This model provided a significant insight on how plants adjust self-growth to adapt to environmental conditions.

## Abbreviations

ABA: Abscisic acid
AFB: Auxin signaling F-Box proteins
ARF: Auxin response factors
GUS: β-glucuronidase
IAA: Indole-3-acetic acid
*OsTAS3a*: *Oryza sativa trans-Acting Short RNA precursor 3a*
PDA: Potato dextrose agar
PEG: Polyethylene glycol
Ta-siRNA: Transacting short interfering RNAs
*TIR1*: *Transport Inhibitor Response 1*
X-Gluc: 5-brom-4-chloro-3-indolyl Glucuronide
